# Diarrhœa of Children

**Published:** 1847-10

**Authors:** 


*Diarrhœa of Children.*—The diarrhœa which accompanies or follows the period of weaning is often fatal; it is not only observable in children who are suddenly deprived of the breast, but also in those who are nursed for too long a period. Dr. Weisse, physician to the Children's Hospital in St. Petersburg, advises the exhibition of raw meat in such cases, and asserts that he has from this practice often derived the most signal advantages. The meat should be hashed, or reduced into a pulp, and two table-spoonfuls may be at first given in four meals.—*Med. Times, from Annales de la Société Med. Chir. de Bruges.*

